# miR-146a-5p Mediates Intermittent Hypoxia-Induced Injury in H9c2 Cells by Targeting XIAP

**DOI:** 10.1155/2019/6581217

**Published:** 2019-05-07

**Authors:** Guofu Lin, Jiefeng Huang, Qingshi Chen, Lida Chen, Dehuai Feng, Shuyi Zhang, Xiaoyun Huang, Yaping Huang, Qichang Lin

**Affiliations:** ^1^Department of Respiratory and Critical Care Medicine, The First Affiliated Hospital of Fujian Medical University, No. 20 Chazhong Road, Taijiang District, Fuzhou 350005, China; ^2^The Second Affiliated Hospital of Fujian Medical University, No. 34 Zhongshan North Road, Licheng District, Quanzhou 362000, China; ^3^Department of Respiratory and Critical Care Medicine, Zhangzhou Affiliated Hospital of Fujian Medical University, No. 59, Shenglixi Road, Xiangcheng District, Zhangzhou 363000, China

## Abstract

MicroRNAs (miRNAs) have emerged as key modulators in the pathophysiologic processes of cardiovascular diseases. However, its function in cardiac injury induced by obstructive sleep apnea (OSA) remains unknown. The aim of the current study was to identify the effect and potential molecular mechanism of miR-146a-5p in intermittent hypoxia(IH)- induced myocardial damage. We exposed H9c2 cells to IH condition; the expression levels of miR-146a-5p were detected by RT-qPCR. Cell viability, cell apoptosis, and the expressions of apoptosis-associated proteins were assessed via Cell Counting Kit-8 (CCK-8), flow cytometry, and western blotting, respectively. Target genes of miR-146a-5p were confirmed by dual-luciferase reporter assay. IH remarkably lowered viability but enhanced cell apoptosis. Concomitantly, the miR-146a-5p expression level was increased in H9c2 cells after IH. Subsequent experiments showed that IH-induced injury was alleviated through miR-146a-5p silence. X-linked inhibitor of apoptosis protein (XIAP) was predicted by bioinformatics analysis and further confirmed as a direct target gene of miR-146a-5p. Surprisingly, the effect of miR-146a-5p inhibition under IH may be reversed by downregulating XIAP expression. In conclusion, our results demonstrated that miR-146a-5p could attenuate viability and promote the apoptosis of H9c2 by targeting XIAP, thus aggravating the H9c2 cell injury induced by IH, which could enhance our understanding of the mechanisms for OSA-associated cardiac injury.

## 1. Introduction

Obstructive sleep apnea (OSA) is part of sleep-associated breathing disorders that are characterized by partial or complete upper airway obstruction during sleep, leading to hypopneas, apneas, repetitive hypoxemia, and recurrent arousals from sleep [1]. Epidemiologic data showed that OSA affects approximately 23.4% of women and 49.7% of men [2]. OSA has been considered an important and independent risk factor for cardiovascular diseases including coronary heart disease, hypertension, and heart failure [3–5]. Intermittent hypoxia (IH) is a critical pathophysiologic mechanism of sleep apnea and the underlying basis for OSA-related heart diseases [6]. A number of studies suggested that IH exposure is related to the increase in myocardial infarction (MI) size [7, 8]. Thus, elucidating the crucial mechanism of preventing IH-related infarction will be helpful to MI therapy.

MicroRNAs (miRNAs) are evolutionally conserved small single-stranded noncoding RNA molecules, which negatively regulate mRNA expression via binding to the 3′UTR of the mRNA [9]. miRNAs play critical roles in cardiac remodelling, including myocardial apoptosis, MI, arrhythmia, and cardiac hypertrophy [1, 10]. For instance, inhibition of miRNA-24 involved in post-MI responses induced cardiomyocytes and fibroblast apoptosis [11]. Overexpression of miR-17-92 resulted in a profound hypertrophic and dilated cardiomyopathy and sudden cardiac death [12]. Recently, miR-146a-5p has been verified as a crucial regulator in the development of numerous cancers such as breast cancer [13], prostate cancer [14], and gastric cancer [15]. Furthermore, miR-146a-5p was upregulated in the myocardial hypoxia/reoxygenation (H/R) cell model and rat model of ischemia/reperfusion (I/R), while troxerutin could exert cardioprotective effects on H/R cells and I/R-injured rats by downregulation of miR-146a-5p [16]. However, the effects and modulatory mechanism of miR-146a-5p in protecting cardiomyocytes from IH-induced injury have not been studied.

In the present study, we exposed H9c2 cells to IH for establishing the in vitro model of myocardial injury. The expression level of miR-146a-5p after IH was detected, and the role of miR-146a-5p dysregulation on IH-induced damage in H9c2 cells was determined by assessing cell viability and apoptosis. Then, we further explored the mechanism of interaction between miR-146a-5p and X-linked inhibitor of apoptosis protein (XIAP), which is a member of the IAP family. It has been reported that XIAP was significantly increased on the I/R animal model [17]. The results of the present study will elaborate the effects of miR-146a-5p in preventing IH-mediated myocardial damage, with the goal of identifying potential options for treatments of OSA-related cardiovascular diseases.

## 2. Materials and Methods

### 2.1. Cell Culture and Establishment of IH Model

H9c2 cell lines were obtained from the Cell Bank of the Chinese Academy of Sciences (Shanghai, China). Cells were grown using Dulbecco's modified Eagle's medium (HyClone) supplemented with 10% fetal bovine serum (Gibco) and 1% penicillin/streptomycin in a humidified atmosphere of a 5% CO_2_ incubator at 37°C (Thermo Fisher Scientific, Waltham, MA, USA). Once H9c2 cells reached 70-80% confluency, IH stimulation was performed as previously described [18], with minor modifications. Cells were carried out under hypoxia condition (repeated cycles of 1% O_2_ with 5% CO_2_ balanced with N_2_ for 35 min) and then normoxic condition (21% O_2_ with 5% CO_2_ balanced with N_2_ for 25 min). Repeated IH exposure was applied for 6 times.

### 2.2. Real-Time Quantitative PCR (RT-qPCR)

After intervention, the mRNA of H9c2 cells was isolated by using a Trizol reagent (Takara) following the manufacturer's protocol. To estimate the expression of miR-146a-5p, the RevertAid™ First Strand cDNA Synthesis Kit (#K1622; Thermo Fisher Scientific) with a special stem-loop primer and SYBR Green PCR Master Mix (#K0223; Thermo Fisher Scientific) were applied to reverse transcription and quantitative PCR. To determine the expression level of XIAP, the One Step SYBR® PrimeScript® PLUS RT-RNA PCR Kit (Takara) was used. U6 and β-actin were used as an internal control. The RT-qPCR was performed on an ABI 7500 thermocycler (Applied Biosystems, Foster City, CA, USA). Relevant primers are listed in Table 1. Fold changes were calculated by the 2^−ΔΔCT^ method.

### 2.3. Cell Transfection

MiR-146a-5p mimics, miR-146a-5p inhibitor, and corresponding scrambled control and small interfering RNA targeting XIAP (si-XIAP) were synthesized by Sangon Biotech Co. (Shanghai, China) and transfected using Lipofectamine 3000 (Invitrogen, USA) following the manufacturer's instructions.

### 2.4. CCK-8 Assay

The cell viability was detected by a Cell Counting Kit-8 (CCK-8; TransGen Biotech, Beijing, China) following the manufacturer's instructions. H9c2 cells were plated in 96-well plates at 5 × 10^3^ cells per well. After IH stimulation, 10 *μ*l solution of CCK-8 was added into each well, and the mixture of 96-well plates was incubated at an incubator for an additional 2 h. The absorbance was measured at 450 nm using a Multiskan GO Spectrophotometer (Thermo Fisher Scientific, USA).

### 2.5. Apoptosis Assay

We assessed cell apoptosis according to double staining with Annexin V-fluorescein isothiocyanate (FITC) and propidium iodide (PI) by flow cytometry analysis. In brief, cardiomyocytes were seeded into 6 well-plates with 1 × 10^5^ cells per well. After IH exposure, cells were washed in phosphate-buffered saline (PBS) and resuspended in 200 *μ*l binding buffer, mixed with 5 *μ*l of Annexin V-FITC and 10 *μ*l of PI, and eventually analyzed by a flow cytometer (Becton Dickinson, USA).

### 2.6. Western Blot Analysis

Proteins were isolated using Mammalian Protein Extraction Reagent (CWBIO, Beijing, China) supplemented with protease inhibitors. The protein concentration was determined by a BCA Protein Assay Kit (CWBIO, Beijing, China). Equal amounts of protein were separated by SDS-PAGE and transferred to PVDF membranes. The membranes were blocked in 5% nonfat dry milk for 1 h and then incubated with primary antibodies at 4°C overnight. After washes, relevant secondary antibodies were used at room temperature for 1 h. Afterwards, the membranes were washed and developed using standard chemiluminescence and the Bio-Rad ChemiDoc™ XRS+System.

### 2.7. Dual-Luciferase Reporter Assay

The pSI-Check2 luciferase reporter vector containing the binding sites of the 3′-UTR of XIAP mRNA or mutant 3′-UTR of XIAP was cotransfected with miR-146a-5p mimics or negative controls into H9c2 cells using Lipofectamine 3000. We measured the luciferase activity by using the luciferase reporter assay kit (Promega) and analyzed it with a luciferase reporter assay system (Promega). Renilla luciferase activities were normalized as control.

### 2.8. Statistics and Data Analysis

All statistical analyses were conducted using SPSS software (version 22.0). All data are presented as the means and standard deviations. Differences were compared by one-way ANOVA, followed by modified Student's *t*-test. The significance was recognized at *P* < 0.05. All experiments were repeated three times.

## 3. Results

### 3.1. IH-Induced Damage in H9c2 Cells

**Figure 1:**
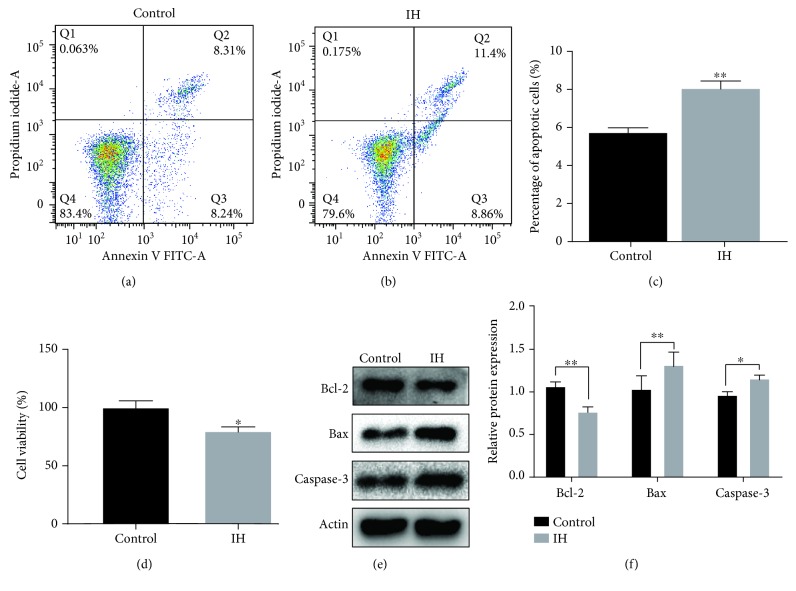
IH suppresses cell viability but contributes to apoptosis in H9c2 cells. (a–c) Cell apoptosis by flow cytometry analysis. (d) Cell viability by a Cell Counting Kit-8. (e, f) Western blotting assays for Bcl-2, Bax, and Caspase-3 protein levels. Relative protein levels are presented as the average expressions normalized to actin. IH: intermittent hypoxia; *n* = 3. (Data are presented as the mean ± SD of three independent experiments. ^∗^*P* < 0.05, ^∗∗^*P* < 0.01, and ^∗∗∗^*P* < 0.001).

To test the role of IH condition for H9c2 cells, cell apoptosis rate and cell viability were assessed under normoxic or IH condition. The results of flow cytometry assay indicated that IH stimulation significantly increased the rate of cell apoptosis (*P* < 0.01; Figures 1(a) and 1(b)). And the results of cell viability showed that there was a significant reduction of cell viability in H9c2 cells under IH condition (*P* < 0.05; Figure 1(c)). Meanwhile, western blot analysis showed that the protein expressions of Bax and Caspase-3 were significantly increased, whereas Bcl-2 protein expression was markedly reduced when compared to the control group (Figures 1(d) and 1(e)).

### 3.2. IH-Induced Changes in the Expression Levels of miR-146a-5p in H9c2 Cells

**Figure 2:**
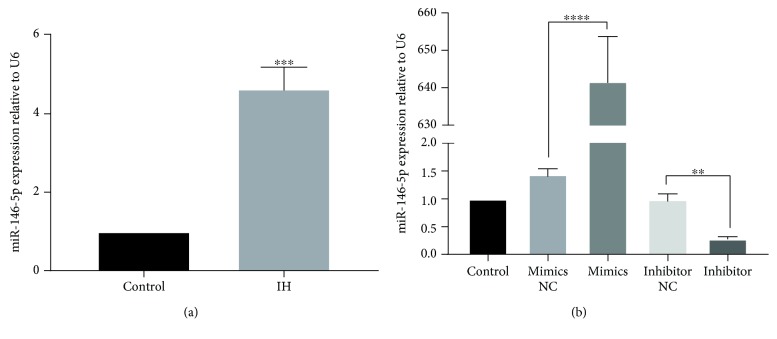
IH causes upregulation of miR-146-5p, and miR-146a-5p is differentially expressed in H9c2 cells after cell transfection. miR-146-5p expression level was evaluated by RT-qPCR. Cells were transfected with miR-146a-5p mimics, miR-146a-5p inhibitor, and corresponding scrambled control. Relative miR-146-5p expression was presented as the average expressions normalized to U6. IH: intermittent hypoxia; *n* = 3. (Data are presented as the mean ± SD of three independent experiments. ^∗^*P* < 0.05, ^∗∗^*P* < 0.01^∗∗∗^*P* < 0.001, and ^∗∗∗∗^*P* < 0001).

**Figure 3:**
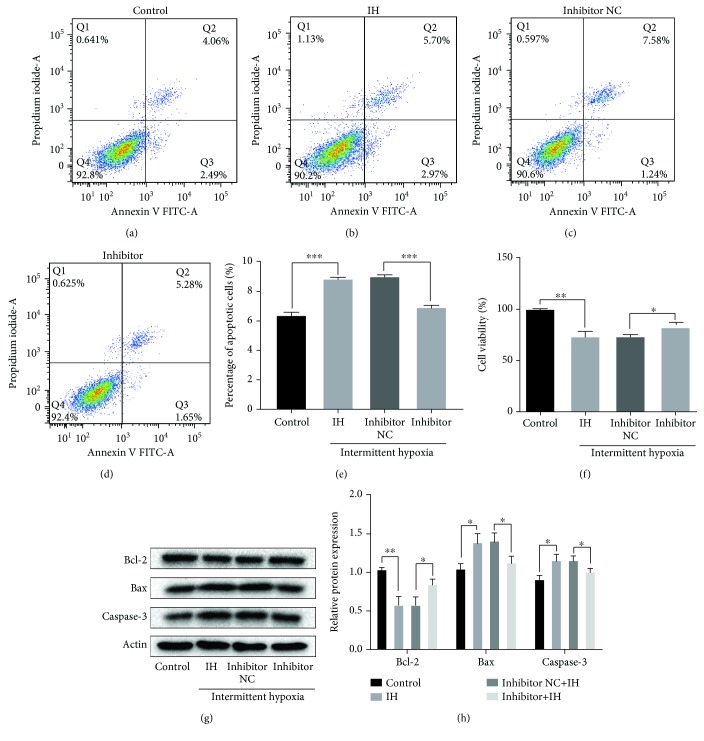
miR-146a-5p silence alleviates IH-induced injury in H9c2 cells. Cells were transfected with miR-146a-5p mimics, miR-146a-5p inhibitor, and corresponding scrambled control. Cells without treatment were acted as control. (a–e) Cell apoptosis by flow cytometry analysis. (f) Cell viability by a Cell Counting Kit-8. (g, h) Expression levels of apoptosis-related proteins by western blot analysis. Relative protein levels are presented as the average expressions normalized to actin. IH: intermittent hypoxia; *n* = 3. (Data are presented as the mean ± SD of three independent experiments. ^∗^*P* < 0.05, ^∗∗^*P* < 0.01, and ^∗∗∗^*P* < 0.001).

To examine the influence of miR-146a-5p in cardiomyocytes, we confirmed the expression levels of miR-146a-5p in IH-mediated H9c2 cells by RT-qPCR. The results showed that miR-146a-5p was significantly upregulated by IH compared to the nontreated cells (*P* < 0.001; Figure 2(a)). To further validate the roles of miR-146a-5p, transfection of H9c2 cells with the miR-146a-5p mimics, miR-146a-5p inhibitor, or corresponding negative control was performed. After transfection, miR-146a-5p expression levels were explored by RT-qPCR. As expected, the expression levels of miR-146a-5p were markedly higher in the miR-146a-5p mimics group compared to those in the negative control group (*P* < 0.0001; Figure 2(b)). The expression levels of miR-146a-5p had a significant reduction after transfecting with the miR-146a-5p inhibitor (*P* < 0.001; Figure 3). These outcomes indicated that the transfection was efficient.

### 3.3. miR-146a-5p Inhibition Alleviates IH-Induced Cell Injury

We performed knockdown experiments to see if the miR-146a-5p inhibitor can protect H9c2 cells from IH-induced injury. Flow cytometry and CCK-8 results indicated that the cell apoptotic rate in the miR-146a-5p inhibitor group was markedly lower compared to that in the negative control group (*P* < 0.001; Figures 3(a)–3(e)), while the cell viability of cardiomyocytes was significantly higher than that of the negative control group after transfecting with the miR-146a-5p inhibitor (*P* < 0.05; Figure 3(f)). In addition, the apoptosis-associated proteins Bcl-2, Bax, and Caspase-3 were tested by western blotting. Western blot analysis showed that IH stimulation significantly upregulated the expression of Bcl-2, whereas it markedly reduced Bax and Caspase-3 protein expressions after transfecting with the miR-146a-5p inhibitor (Figures 3(g) and 3(h)).

### 3.4. miR-146a-5p Negatively Regulates Expression of XIAP, and XIAP Is Confirmed as a Direct Target Gene of miR-146a-5p

**Figure 4:**
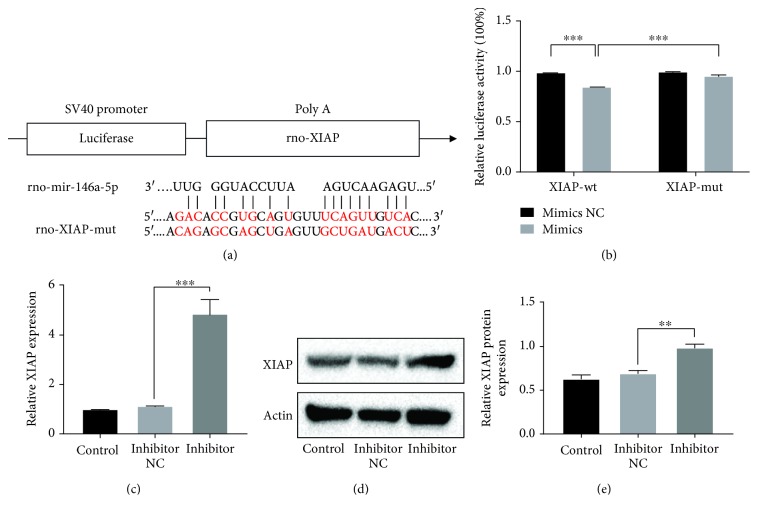
XIAP is a target gene of miR-146a-5p, and XIAP expression could be negatively regulated by miR-146a-5p in H9c2 cells. (a) The putative binding site for miR-146a-5p in the 3′-UTR of XIAP mRNA. (b) Luciferase reporter assay. Cells were cotransfected with wild-type or mutant XIAP 3′-UTR reporters and miR-146a-5p mimics or corresponding control. (c–e) H9c2 cells were transfected with miR-146a-5p mimics or corresponding control. mRNA and protein expressions of XIAP were analyzed by western blot analysis. *n* = 3. (Data are presented as the mean ± SD of three independent experiments. ^∗^*P* < 0.05, ^∗∗^*P* < 0.01, and ^∗∗∗^*P* < 0.001).

We performed bioinformatic analysis to investigate the potential mechanism by which miR-146a-5p inhibition suppressed IH-induced cell injury. Using RNAhybird, miRbase, and TargetScan, XIAP was predicted as a new target gene for miR-146a-5p. The binding sites of the XIAP 3′UTR and miR-146a-5p are shown in Figure 4(a). A dual-luciferase reporter assay was carried out to confirm whether miR-146a-5p directly targeted the XIAP 3′UTR. The results showed that luciferase activity was markedly decreased in cardiomyocytes cotransfected with miR-146a-5p mimics and XIAP-WT compared to that of cotransfection with mimics control and XIAP-WT (*P* < 0.001; Figure 4(b)). In addition, the results showed expressions of XIAP at the levels of mRNA and protein were markedly increased by knocking down miR-146a-5p when compared to the negative control group (Figures 4(c)–4(e)). Overall, these results indicated that XIAP is a direct target gene of miR-146a-5p.

### 3.5. Knockdown of XIAP Abolished the Protective Effects of miR-146a-5p Inhibition against IH-Induced Injury in H9C2 Cells

**Figure 5:**
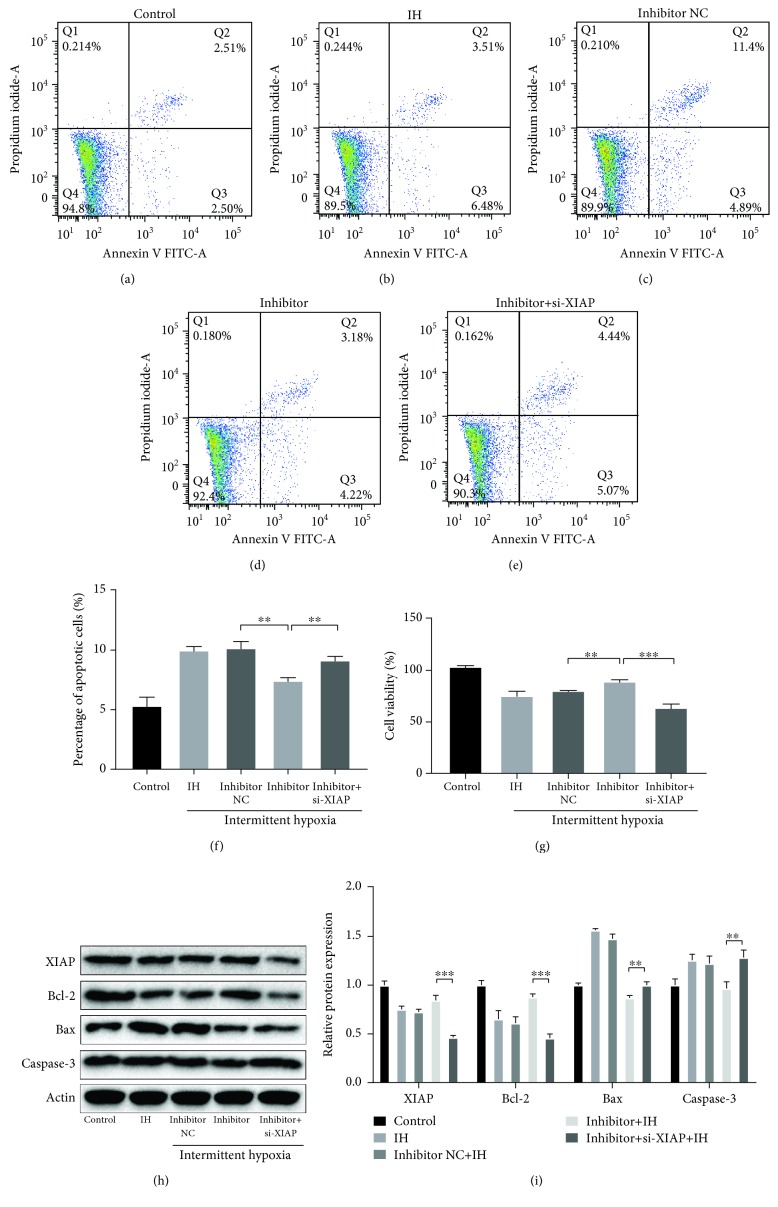
The effects of miR-146a-5p inhibition in H9c2 cells under IH condition are reversed by XIAP knockdown. miR-146a-5p inhibitor, corresponding scrambled control, and small interfering RNA targeting XIAP (si-XIAP) were transfected into H9c2 cells. Cells without transfection were acted as control. (a–f) Cell apoptosis by flow cytometry analysis. (g) Cell viability by a Cell Counting Kit-8. (h, i) Western blotting assays for XIAP, Bcl-2, Bax, and Caspase-3 protein expressions. Relative protein levels are presented as the average expressions normalized to actin. IH: intermittent hypoxia; *n* = 3. (Data are presented as the mean ± SD of three independent experiments. ^∗^*P* < 0.05, ^∗∗^*P* < 0.01, and ^∗∗∗^*P* < 0.001).

Next, we validated if XIAP is related to the effects of miR-146a-5p IH-induced injury. H9c2 cells were transfected with si-XIAP, miR-146a-5p inhibitor, or corresponding negative control. As shown in Figures 5(a)–5(g), the effects of miR-146a-5p silence on cell viability, cell apoptotic rate, and expression levels of apoptosis-related proteins were all reversed through XIAP knockdown compared to the negative control group under IH condition (Figures 5(h) and 5(i)). Thus, we can conclude that miR-146a-5p inhibition may attenuate IH-mediated cell injury through upregulating XIAP.

## 4. Discussion

In the current study, our data demonstrated that IH-induced H9c2 cell injury and miR-146a-5p were markedly increased by IH condition. But the miR-146a-5p inhibitor can prevent H9c2 cells from IH-induced damage, as evidenced by the improved cell viability, the reduced apoptotic rate, the downregulated Bax, Caspase-3, and the increased Bcl-2. Afterwards, miR-146a-5p was validated to negatively regulate XIAP and XIAP was further confirmed as a direct target gene of miR-146a-5p by luciferase reporter assay. Furthermore, roles of miR-146a-5p suppression in H9c2 cells could be relieved through downregulating XIAP expression.

OSA is a breath disease characterized by recurrent episodes of upper airway obstruction and subsequent IH during sleep, which is considered an independent risk factor for cardiac diseases, including myocardial infarction, hypertension, and stroke. Previous studies showed that IH exposure markedly increased numbers of TUNEL-positive cardiomyocytes, inducing cardiac remodelling by oxidative stress and inflammatory response [19]. It was reported that adipocytes originating from human showed strong sensitivity to inflammatory gene expression under IH condition, including nuclear factor-*κ*B (NF-*κ*B), TNF-alpha, interleukin (IL)-8, and IL-6 [20]. Additionally, oxidative stress induced by IH appears to mediate the deleterious cardiovascular effects and increase myocardial susceptibility to infarction [21]. Similarly, animal experiments showed IH significantly enhanced I/R-induced myocardial injury and increased sensibility to myocardial infarction [22]. In our study, IH stimulation markedly reduced cell viability, increased cell apoptotic rate, and changed the expression levels of apoptosis-associated proteins in H9c2 cells. Therefore, how to relieve IH-related myocardial injury is a growing concern.

Several miRNAs were verified to play a potential role in regulating I/R-induced injury in H9c2 cells. For example, miR-192-5p was upregulated after I/R and knockdown of miR-192-5p alleviated I/R-induced apoptotic death in cardiomyocytes [23]. Suppression of miR-122 could relieve I/R-induced cardiomyocyte injury by upregulating the expression of GATA-4 [24]. Overexpression of MLK3 diminished the impact of miR-140-5p inhibition in H9c2 cells under I/R condition, as it markedly decreased cell viability and changed apoptosis-related proteins, which suggested miR-140-5p inhibition of I/R-induced cell injury by downregulating MLK3 expression [25]. However, only a few studies focused on the effects of miRNAs in regulating cardiovascular injury under IH condition. It was reported that the miR-193 inhibitor could reverse IH-induced apoptosis and autophagy relative protein expression in mouse aortic endothelial cells [26]. Recently, downregulation of miR-30a seemed to enhance IH-associated endothelial cell autophagy by increasing Beclin-1 [27]. Our results showed miR-146a-5p was markedly increased after IH; therefore, we chose miR-146a-5p for this study and investigated the relationship between miR-146a-5p and myocardial injury under IH condition.

Growing evidence suggests that the expression levels of miR-146a-5p are relevant to inflammation [28], coagulation [29], anticancer [13], proliferation, and apoptosis [30]. Based on these pathophysiologic effects, miR-146a-5p has been evidenced to mediate cytoprotection and organ protection. In our present study, miR-146a-5p was markedly upregulated by IH, whereas the viability and apoptosis of H9c2 cells were relieved after transfecting with the miR-146a-5p inhibitor. Similarly, it was reported that miR-146a-5p expression was associated with increased expression of inflammatory genes TLR4, NF-*κ*B, IL-6, and TNF-*α* in mononuclear leukocytes [31]. Suppression of miR-146a-5p in mesenchymal stem cells (MSCs) inhibited their proliferation but promoted their migration, suggesting that miR-146a-5p is crucial to uncouple the direct effects of proliferation and motility of MSCs [32]. Our study together with several previous studies [16, 30] suggested that miR-146a-5p also could mediate IH-induced injury in H9c2 cells.

To investigate the underlying mechanism of miR-146a-5p dysregulation in IH-mediated injury in H9c2 cells, we performed bioinformatic analysis and dual-luciferase reporter assay. It is well known that miRNAs have important impacts on various biological processes by modulating the expression of their target genes [33]. XIAP, one of the inhibitors of apoptosis (IAP) family members, has been confirmed as an important regulator of cell apoptosis [34]. In addition, miR-146a-5p was predicted to target XIAP 3′UTR and studies between miR-146a-5p and XIAP have not been investigated. In this study, the luciferase reporter gene assay-validated XIAP was a direct target of miR-146a-5p and XIAP expression can be negatively modulated by miR-146a-5p. Interestingly, a previous study indicated that XIAP overexpression was prevented from activation of pathological caspase activation and tissue loss after hypoxic-ischemic (HI) brain injury [35]. XIAP may be concerned with I/R-mediated cardiac injury. In some ways, overexpression of XIAP could decrease both myocardial apoptosis and infarction under I/R condition, which attributed to the ability of XIAP to inhibit Caspase-3 [36]. Moreover, decreased miR-181a expression could suppress apoptosis of cardiomyocytes via targeting XIAP to increase Bcl-2 and downregulate Bax expression [37]. Considering the potential modulatory relationship between miR-146a-5p and XIAP, we anticipate that miR-146a-5p may regulate IH-mediated injury in H9c2 cells by modulating XIAP expression. Indeed, our current study demonstrated that XIAP knockdown reversed the effects of miR-146a-5p inhibition on cell viability, cell apoptotic rate, and expressions of apoptosis-related proteins. Together, these results demonstrated that miR-146a-5p aggravated IH-induced injury by XIAP in H9c2 cells.

The aim of the current study was only to evaluate the effect and potential mechanism of miR-146a-5p downregulation in vitro experiments as an initial exploration. However, we must acknowledge that there were some limitations in our study. Different stimulation times of IH may have different effects on H9c2 cells, which needs to be further verified. Moreover, H9c2 is a cardiomyocyte line derived from a rat embryo. Considering that the OSA often occurs in adult patients, more in vitro experiments using adult rat cardiomyoblast and experimental animal models are still needed for further study in the future.

## 5. Conclusions

In summary, our present study confirmed that miR-146a-5p was increased in H9c2 cells under IH condition and miR-146a-5p inhibition could protect H9c2 cells from IH-induced injury. Moreover, miR-146a-5p mediates IH-induced cell injury by regulating XIAP expression. Our findings will contribute to the development of a therapeutic strategy for OSA-associated cardiac diseases.

## Figures and Tables

**Table 1:** Sequence information.

	Sequence (5′-3′)
Special stem-loop primer of miR-146a-5p	GTCGTATCCAGTGCGTGTCGTGGAGTCGGCAA- -TTGCACTGGATACGACAACCCAT
miR-146a-5p	Sense: GGGGTGAGAACTGAATTCCAT
	Antisense: CAGTGCGTGTCGTGGAGT
miR-146a-5p mimics	Sense: UGAGAACUGAAUUCCAUGGGUU
	Antisense: CCCAUGGAAUUCAGUUCUCAUU
Mimic control	Sense: UUCUCCGAACGUGUCACGUTT
	Antisense: ACGUGACACGUUCGGAGAATT
miR-146a-5p inhibitor	AACCCAUGGAAUUCAGUUCUCA
Inhibitor control	CAGUACUUUUGUGUAGUACAA
XIAP	Sense: GGTGCAAGAAGCTATACGAATGG
	Antisense: AGTTGCTCCCAGATGTTTGGAG
si-XIAP	Sense: GCCAGACUAUGCCCAUUUATT
	Antisense: UAAAUGGGCAUAGUCUGGCTT
U6	Sense: CTCGCTTCGGCAGCACA
	Antisense: AACGCTTCACGAATTTGCGT
*β*-Actin	Sense: CGAGTACAACCTTCTTGCAGC
	Antisense: ACCCATACCCACCATCACAC

## Data Availability

The data used to support the findings of this study are included within the article.
